# Efficacy of Advanced Odomos repellent cream (N, N-diethyl-benzamide) against mosquito vectors

**Published:** 2011-04

**Authors:** P.K. Mittal, U. Sreehari, R.K. Razdan, A.P. Dash, M.A. Ansari

**Affiliations:** National Institute of Malaria Research (ICMR), New Delhi, India; * *WHO-SEARO, New Delhi, India*; ** *Regional Medical Research Centre for Tribals (ICMR), Jabalpur, India*

**Keywords:** Advanced odomos, *Anopheles culicifacies*, *An. stephensi*, *Aedes aegypti*, DEET, repellency

## Abstract

**Background & objectives:**

Repellents are commonly used personal protection measures to avoid mosquito bites. In the present study, Advanced Odomos cream (12% N, N-diethyl-benzamide) was tested for its efficacy against mosquitoes in comparison to DEET (N,N-diethyl-3-methyl benzamide).

**Methods:**

Bioassays were conducted to assess the repellency of Advanced Odomos and DEET creams against *Anopheles stephensi* and *Aedes aegypti*. Their efficacy was tested on human volunteers applied with different concentrations of test creams ranging from 1 to 12 mg/cm^2^ and by exposing them to mosquitoes at hourly intervals. Field evaluation was also carried out to test the duration of protection of the test creams against *Anopheles* and *Aedes* mosquitoes during whole night and day time collections, respectively on human volunteers. Mosquito collections were done using torch light and aspirator.

**Results:**

Complete (100%) protection was achieved at 10 mg/cm^2^ cream formulation of Advanced Odomos (1.2 mg a.i/cm^2^) dose against *An. stephensi* and 12 mg/cm^2^ (1.44 mg a.i./cm^2^) against *Ae. aegypti* on human baits. There was no statistically significant differences in per cent protection against mosquito bites between Advanced o0 domos and DEET cream (*P*>0.05) in respective doses. Complete protection up to 11 h was observed against *Anopheles* mosquitoes during whole night collections and up to 6 h against *Ae. aegypti* in day time collections. No adverse reactions such as itching, irritation, vomiting, nausea, *etc*. were reported by the volunteers.

**Interpretation & conclusions:**

Advanced odomos cream applied at 10 mg/cm^2^ concentration provided 100% protection from *Anopheles* mosquitoes up to 11 h whereas about 6 h protection was recorded against *Ae. aegypti*. The laboratory and field trials indicate that for longer protection against Anopheles mosquitoes 10 mg/cm^2^ will be appropriate and in case of *Ae. aegypti* more than 10 mg/cm^2^ application is required for complete protection. In conclusion, the Advanced Odomos cream was comparable to the known repellent cream DEET for prolonged protection against malaria and dengue vectors.

Several vector control measures such as chemical, biological, environmental and personal protection measures are taken to prevent transmission of malaria, dengue/dengue haemorrhagic fever and other mosquito-borne diseases. Personal protection is one of the established methods to prevent mosquito bites. Use of household mosquito repellents by individuals and communities also play an important role particularly against day-time biting mosquito species such as *Aedes*, the vector of dengue, dengue haemorrhagic fever and chikungunya and during dusk time against other mosquitoes including malaria vectors^1^. Repellents that are currently available are either synthetic chemicals, such as DEET (N,N-diethyl-3-methyl benzamide, previously called N,N-diethyl-m-toluamide), or plant derived chemicals such as Citronella. DEET is the most effective and best studied insect repellent currently in the market^1^–^6^. Although DEET is very good repellent, the non-availability of 3-methylbenzoic acid from indigenous sources has made it expensive chemical in India^7^. The alternative, dimethylphthalate (DMP), also need to be replaced in India because of its low efficacy^7^. In view of this, significant efforts have been made toward developing longer-lasting and/or more cosmetically accepted repellent products. In the present study, Advanced Odomos cream developed by M/s. Balsara Home Products Ltd. (now M/s. Dabur Research Foundation, Sahibabad, UP) was evaluated for its efficacy under laboratory and field conditions against *Anopheles* and *Aedes* mosquitoes. DEET was taken as gold standard for comparison.

## Material & Methods

Advanced Odomos cream (12% N, N-diethyl-benzamide) and DEET cream (12% N, N-diethyl-3-methyl benzamide) supplied by M/s. Balsara Home Products Ltd. Mumbai, India (now Dabur Research Foundation) were used in the present evaluation. The study was undertaken during December 2005 to November 2006 in the laboratory and in field conditions of the respective habitats of *Anopheles* spp and *Aedes aegypti* as per guidelines prescribed in ‘Protocols for uniform evaluation of insecticides for use in vector control’^8^ with some modifications. The institutional Human Ethical Committee approved the study.

*Laboratory tests on human baits*: The tests were conducted at the National Institute of Malaria Research, New Delhi, in a laboratory maintained at 27 ± 2°C and 60-70 per cent relative humidity. The light intensity was regulated at 300–500 lux for testing against laboratory colonized *Ae. aegypti*, a day biting mosquito and at about 20–50 lux for *An. stephensi*, a night biter. Laboratory reared 3–5 day old female mosquitoes starved for overnight were used for bioassays. Testing was done in the laboratory maintained at standard temperature and humidity against laboratory colonized strains of *An. stephensi* and *Ae. aegypti* in cage bioassays using human volunteers. Repellent cream was applied on an approximate 100 cm^2^ area of one of the fore arm (wrist to elbow) of human volunteer. Remaining exposed area was covered with a cloth sleeve. Different concentrations — 4, 8, 10, and 12 mg/cm^2^ of whole cream base (0.48, 0.96, 1.2 and 1.44 mg/cm^2^ active ingredient) were tested. Tests were continued on each species until 100 per cent protection was obtained in four hours of exposure. Exposures of hand and arm applied with different doses of the repellent cream and control were made separately in different cloth cages containing 100 three-day old mosquitoes of each species that were pre-starved for overnight. Inserting an untreated hand into the cage before the start of each exposure ensured the propensity of mosquito for biting. Hands of the volunteer treated with different concentrations of Advanced odomos cream, DEET cream and a base cream without active ingredient (control) were introduced in different cages. Five minute landing counts were made manually at 0, 1, 2, 3 and 4 hours. Volunteers were asked to remove treated hands between the exposure intervals. Number of mosquitoes landing on different doses of repellent cream and control at different intervals was scored manually. The experiment was replicated four times for each concentration of the test cream and controls. Per cent protection for each concentration and species was calculated using the formula mentioned below. The time between the initial introduction of repellent treated hand and the first confirmed landing followed by the second successive landing was recorded as the protection time and the test was terminated after the second landing of the mosquito.

$$\mathrm{Per}\;\mathrm{cent}\;\mathrm{protection}\;=\frac{No.\;landing\;on\;negative\;control\;-\;No.\;landing\;on\;treated\;with\;repellent}{No.\;landing\;on\;negative\;control}\times 100$$

*Field evaluation*: Field trials with Advanced Odomos and DEET creams against malaria vectors *An. stephensi, An. culicifacies* and other mosquito spp including common pest mosquito *Culex quinquefasciatus* were undertaken in Pacheria village in Loni PHC, District Ghaziabad, Uttar Pradesh, India. The village is situated on the bank of the Yamuna river and is endemic for malaria where *An. culicifacies* and *An. stephensi* are principal malaria vectors. The field tests were conducted from 1900 to 0600 h against *Anopheles* mosquitoes. Field trials against day-biting *Ae. aegypti* mosquitoes were carried out during day time from 0700 to 1800 h in hutments of Railway Colony, Badarpur, Delhi, India.

Four night /day collections were made during July to September 2006. In the village/locality, six houses were selected, two each for Advanced Odomos, DEET and control (cream without active ingredient). The houses and volunteers were selected randomly and volunteers (baits) as well as insect collectors were blinded for the doses and repellent creams. Pre-informed and free consent was obtained before hand from the volunteers participating as baits. The cream was applied on exposed parts as per the specified dose (face and legs below the knee to ankle) and the remaining parts were covered with thick clothes. The volunteers were allowed to relax on cot throughout the night. One insect collector covered completely with clothes, gloves and head mask was deployed to collect mosquitoes landing on the volunteer. The mosquitoes landing on the bait were collected before engorging. In addition, mosquitoes that entered the room having bait and resting inside were also collected. The insect collector was rotated after every two hours.

Mosquitoes collected on baits were identified into species using standard keys in the laboratory^9^–^10^. The time between application of repellent and the second successive landing on the bait was recorded as the protection time and the average of eight replicates was calculated as the mean protection time. The per cent protection data were transformed using arcsine and subjected to statistical analysis to find out the differences between means at 0.05 per cent level of significance between Advanced Odomos and DEET creams. The data were compared using Student t-test for differences between means at 0.05 per cent level of significance.

## Results

Laboratory evaluation of Advanced Odomos and DEET creams against *An. stephensi* and *Ae. aegypti* on human baits is shown in Table I. On human baits, 4, 8, and 10 mg/cm^2^ doses were tested against *An. stephensi* and in case of *Ae. aegypti* 12 mg/cm^2^ dose was also tested additionally. 100 per cent protection was achieved at 10 mg/cm^2^ dose against *An. stephensi* and 12 mg/cm^2^ against *Ae. aegypti* up to 4 h of observation. There was no statistically significant differences in per cent protection against mosquito bites between Advanced Odomos and DEET creams in respective doses.

**Table I:** Per cent protection against mosquito species exposed to human volunteers applied with Advanced Odomos and DEET creams in cage bioassays in laboratory conditions

Dose mg/cm^2^	Per cent protection			
	*An. stephensi*		*Ae. aegypti*	
	Advanced Odomos	DEET	Advanced Odomos	DEET
4	84.7 ± 0.8 (1)	88 ± 0.7 (2)	76.1 ± 1.8 (0)	86.9 ± 0.93 (0)
8	95.5 ± 1.1 (2)	97.0 ± 0.7 (4)	87.5 ± 0.7 (2)	90.2 ± 1 (2)
10	100 ± 0 (>4)	100 ± 0 (>4)	96.5 ± 2.5 (4)	97.2 ± 0.82 (4)
12	ND	ND	100(>4)	100 (>4)

Values are mean ± SD of four replicate tests. Figures in parentheses are duration of protection in hours (the time at which second confirmatory bite was recorded), ND, not done

Results of field trials of Advanced Odomos and DEET creams during whole night bait collections against different species of mosquitoes are shown in Table II. Per cent protection with Advanced odomos cream applied at 10 mg/cm^2^ was 100 per cent against *An. culicifacies, An. stephensi, An. anuularis* and *An. subpictus*; and 98.8 per cent against *Cx. quinquefasciatus* up to 11 h of observation time. The per cent protection with DEET cream applied at the same dosages was 100 per cent against all the above mentioned species.

**Table II:** Field evaluation of Advanced Odomos and DEET creams (10 mg/cm^2^) against mosquitoes on human baits

Mosquito Species	Intervention	Total no. collected	No. landed on baits	Per cent protection	Average protection time (h)
*An. culicifacies*	Odomos	43	0	100 ± 0	11
	DEET	45	0	100 ± 0	11
	Control	79	37		
*An. stepehensi*	Odomos	87	0	100 ± 0	11
	DEET	92	0	100 ± 0	11
	Control	141	51		
*An. annularis*	Odomos	60	0	100 ± 0	11
	DEET	57	0	100 ± 0	11
	Control	101	26		
*An. subpictus*	Odomos	248	0	100 ± 0	11
	DEET	284	0	100 ± 0	11
	Control	360	74		
*Cx. quinquefasciatus*	Odomos	370	2	98.8 ± 0.25	9
	DEET	390	0	100 ± 0	11
	Control	483	172		
Aedes aegypti*	Odomos	92	4	92.5 ± 2.4	6.2 ± 0.4
	DEET	78	2	96.2 ± 1.1	6.75 ± 0.2
	Control	176	54		

Data of eight replicates.

* Day time collections were made in case of *Ae. aegypti* from 0700 to 1800 h

The results of field trials against *Ae. aegypti* showed 92.5 per cent protection (repellency effect) with Advanced Odomos cream and 96.2 per cent protection with DEET cream applied at 10 mg/cm^2^. Results further revealed that Odomos cream provided protection up to 6.2 ± 0.4 h and DEET cream provided protection up to 6.75 ± 0.2 h.

All the volunteers were provided with a questionnaire regarding their opinion on protection from mosquito bites and side-effects, if any, such as itching or other aberrations of the skin and they did not report any side effects of application of both creams.

## Discussion

A number of laboratories are screening different compounds of different nature and origin for mosquito repellent properties with the aim of developing a formulation giving similar or better protection than DEET, which is used since 1950s. Although effective, DEET is not the ideal product, as allergic and side effects have been reported^11^ and its solvents can damage plastics and other synthetic materials. Osimitz and Grothaus^12^ concluded that ‘DEET can be used with the confidence that the risk of serious adverse effects is very low’. The controlled -release insect repellent formulations for topical use that will provide extended protection against biting arthropods, be safe and pleasant to use and be compatible with plastics, synthetic fabrics and similar materials are most important for prolonged protection against bites of arthropod insects.

In laboratory tests, both DEET and Advanced Odomos creams provided 100 per cent protection when applied at 10 mg/cm ^2^dose against *An. stephensi* and at 12 mg/ cm^2^ against *Ae. aegypti*. In field tests on human volunteers, both creams provided complete protection up to 11 h against *Anopheles* mosquitoes and about 6 h against *Ae. aegypti*. The present study results are in conformity with earlier reports. In one laboratory study carried out elsewhere^13^, 50 per cent DEET provided up to 4 h protection against *Ae. aegypti* mosquitoes and increasing the concentration to 100 per cent provided only one hour additional protection. In another study^14^, 12.5 per cent DEET provided over 6 h of protection against Ae. albopictus; doubling DEET concentration to 25 per cent increased the protection time only to about 8 h. Frances *et al*^15^ reported contrasting results between laboratory and filed efficacy of repellent formulations against *Anopheles* mosquitoes. Another study^16^ reported that three brands of DEET formulations provided >99 per cent protection from bites of *Ae. taeniorhyncus* in field conditions and >95 per cent protection against *An. quadrimaculatus* in cage studies. Kalyanasundaram & Mathew^2^ reported about 6- 6.5 h of protection with diethylbenzamide against *Aedes aegypti*. In the present study also a protection time of about 6 h was observed with Advanced Odomos cream against *Ae. aegypti*. In conclusion, the present study results demonstrated that the efficacy of Advanced Odomos and DEET creams was comparable in providing protection against mosquito bites for longer duration.
